# Assessment of the hematological profile of children with chronic kidney disease on follow-up at St. Paul’s Hospital Millennium Medical College and Tikur Anbessa Specialized Hospital in Addis Ababa, Ethiopia

**DOI:** 10.1186/s12882-024-03464-7

**Published:** 2024-01-30

**Authors:** Melaku Shenkut, Fekadu Urgessa, Rahel Alemu, Bezaye Abebe

**Affiliations:** 1 Department of Medical Laboratory Sciences, College of Health Science, Addis Ababa University, Addis Ababa, Ethiopia; 2 Department of pediatrics and child health, College of Health Science, Addis Ababa University, Addis Ababa, Ethiopia

**Keywords:** Children, Hematological profile, Chronic renal failure

## Abstract

**Background:**

Chronic kidney disease (CKD) is a major public health issue with an increasing incidence and prevalence worldwide. In CKD, hematological parameters are influenced, and the effect increases with CKD stage. Thus, the aim of this study was to assess hematological profile of children with CKD on follow up at Tikur Anbessa Specialized Hospital and St. Paul’s Hospital Millennium Medical College in Addis Ababa, Ethiopia.

**Methods:**

A cross-sectional study was carried out from March 1 to June 30, 2021 among 238 children with CKD. EDTA tubes were used to collect 4 ml blood samples, which were then examined by Beckman Coulter automated hematology analyzer. SPSS Version 20 was used for statistical analysis, and a bivariate and multivariate regression model were applied to assess correlations. Mean and standard deviation was used to determine hematological profiles.

**Results:**

The total number of patients in the study were 238, with 42 (59.7%) of them being men. The majority of the patients (81%) had CKD stage 1. Mean ± standard deviation determined for white blood cell (WBC) parameters in (thousand/µL); WBC, Neutrophil, Lymphocytes, Eosinophil, Monocytes and Basophil were 8.93 ± 3.32, 4.6 ± 8.31, 2.79 ± 1.62, 0.31 ± 0.51, 0.50 ± 3.03 and 0.03 ± 0.24, respectively. For some of red blood cell (RBC) parameters; RBC (million/ µL), Hemoglobin (Hgb) (g/dL), Hematocrit (Hct) (%) and Mean cell volume (fl.) were 4.73 ± 0.87, 12.82 ± 2.76, 38.28 ± 7.53 and 80.32 ± 7.89, respectively. For the platelet count (PLT) (thousand/µL) and Mean Platelet volume (MPV) (fL) 349.34 ± 130.18 and 9.03 ± 4.31 were determined, respectively. This study also found hematologic parameters such as RBC, HGB, HCT and MPV were found to be positively correlated with eGFR with a *P*-value < 0.05 for all parameters.

**Conclusion:**

The study found that the majority of study participants were in stages 1 to 3 based on their estimated glomerular filtration rate (eGFR). Some of hematological parameters found to have positive correlation with eGFR. There is a need to improve multiple aspects of CKD management, including routine hematological tests for children with chronic kidney disease.

## Introduction

Chronic kidney disease (CKD) is a major public health issue worldwide. It is defined by persistent urine abnormalities, structural abnormalities, or impaired excretory renal function suggestive of a loss of functional nephrons [1]. Although CKD is defined by the presence of kidney damage or an estimated glomerular filtration rate (eGFR) less than 60 ml/min/1.73 m^2^, it can last for 3 months or longer, regardless of the cause. Chronic kidney disease (CKD) is recognized as a major non-communicable disease with a growing epidemic global impact [2].

Chronic kidney disease (CKD), also known as Kidney or renal failure, refers to all five stages of kidney damage, beginning with very mild damage in stage 1 and progressing to complete kidney failure in stage 5. eGFR values were used for CKD staging in children over the age of two because GFR values in younger children were low due to on-going renal maturation [3]. Congenital kidney abnormalities, glomerulonephritis, systemic disease, hereditary, sepsis, and other rarer cases, such as drug (toxin)-related renal disease, were the causes of CKD in children. CKD can be caused by any of three disease processes: pre-renal (lower renal perfusion pressure), intrinsic renal (pathology of the vessels, glomeruli, or tubules-interstitial), or post-renal (obstructive). Anemia is the most common complication of chronic kidney disease, and it can develop in CKD patients due to low hemoglobin concentration and hematocrit.

Hematological parameters such as white blood cell (WBC), platelet (PLT), and red blood cell (RBC) parameters are usually affected in CKD. These are common in CKD due to erythropoietin (EPO) deficiency/reduction and other factors such as increased hemolysis, suppression of bone marrow erythropoiesis, hematuria, and gastro intestinal blood loss. CKD appears late in End-stage renal disease (ESRD) and is the terminal stage of chronic renal failure with irreversible progressive deterioration in renal function [4]. However, compared to the general population, children with CKD continue to have very high rates of morbidity and mortality. Cardiovascular disease and infections were the leading causes of death in children with CKD, accounting for 30–40% of deaths [5].

Chronic kidney disease (CKD) is a global public health issue, with rising healthcare costs, particularly in developing countries such as Ethiopia. Its burden is epidemic, affecting both developed and developing countries. According to the global burden of disease 2015 study, CKD was the 17th leading cause of death worldwide in 2015 and one of the fastest growing major causes of death [1]. Lack of advanced diagnostic infrastructure, treatment facilities, and human resources, particularly in Africa, frequently leads to inaccurate diagnosis and suboptimal treatment of children with renal diseases.

There had never been any research done in Ethiopia. In order to better understand the hematological profile of children with chronic kidney disease, this study follow them as they receive follow-up care at Tikur Anbessa Specialized Hospital (TASH) and St. Paul’s Hospital Millennium Medical College (SPHMMC) in Addis Ababa, Ethiopia. The study’s data findings could be useful in preventing unnecessary complications, enhancing the quality of life for people with chronic renal diseases, and extending their life expectancies. The information from this study was helpful for decision-makers in the planning, implementation, and evaluation of various interventions related to improving the care provided in the follow-up set up as well as health care workers who work in follow-up units through identification and improving the service quality.

## Methods

A cross-sectional study was carried out from March 1 to June 30 2021 at TASH and SPHMMC in Addis Ababa, Ethiopia. These two sites are the only governmental facilities provide advanced treatment for CKD patients. The study population consisted of all children with chronic kidney diseases who were enrolled in the follow-up program at SPHMMC and TASH in Addis Ababa City during the research period. The convenient sampling technique was applied to consider all children available during the study period. As a result, the study consists 20 children from SPHMMC and 218 from TASH that makes total sample size 238.

To collect socio-demographic and other related data, a pre-designed questionnaire by authors was used. Aseptically, 4 mL of blood was collected into a lavender-top vacationer tube containing K2-Ethylene diamine tetra acetic acid. To avoid blood clot formation, the blood was gently mixed with EDTA anticoagulant. All venous blood samples were analyzed within 24 h of being collected. Anthropometric measurements like height, weight and MUAC were also determined during sample collection. The stages of CKD were determined by calculating the estimated glomerular filtration rate using the modified Schwartz formula of 0.413*Ht(cm)/(Serum creatinine in mg/dl).

To get hematological parameters all samples were analysis using Beckman Coulter’s automated hematology analyzer. The DxH 800 analyzes 24 parameters quickly, including a 5-part WBC differential and histograms for RBC, PLT, and WBC in blood. The Coulter Principle underpins the DxH 800 CBC analysis. In whole blood mode, this device can analyze factors such as reticulocyte count with peripheral morphology, WBC, RBC, HGB, HCT, MCV, MCH, MCHC, NEUT %, LYM %, PLT, LYM #, NEU #, MPV, RDW-CV and RDW-SD, and P-LCR.

To keep data quality, all pre-analytical and analytical variables that can influence test results must be reflected in analytical results from reference populations. As a result, all pre-analytical factors were carefully defined and used for testing, including subject preparation, sample collection and processing, the analytical method, and instrumentation (daily, weekly, and monthly preventive maintenance) check-ups. The results of the entire CBC were recorded, handled appropriately, and secured.

### Data analysis and interpretation

SPSS Version 20 statistical software was used to enter and analyze the descriptive data. Mean and standard deviation was used to determine the hematological parameters among children with CKD. To identify factors that have correlated with the dependent variable; Pearson correlation was performed. *P*-value < 0.05 cut off was used to assess significance for correlation of hematological parameters with different factors.

## Results

### Socio-demographic characteristics

 A total of 238 children with chronic kidney disease were included in the study. Of them 142 (59.7%) of these were men whereas 176 (73.9%) of the children were over the age of five (5–17 years). Children in this study had a mean weight of 24.25 ± 10.97 kg, a mean height of 1.24 ± 0.257 cm, a mean MUAC of 16.43 ± 4.187, and a mean BMI of 15.23 ± 2.94. In terms of education, 152 (63.9%) of the patients have a primary education. About 77 (32.4%) of patients with CKD had been suffering from the disease for 1 to 3 years (Table 1).

**Table 1:** Socio demographic characteristics of children with chronic kidney disease on follow up in SPHMMC and TASH, Addis Ababa (*n*=238)

Socio-demographic character	Variable		Frequency	Percent
Sex	Male		142	59.7%
	Female		96	40.3%
Age	1-3 years		33	13.9%
	3-5years		29	12.2%
	> 5 years		176	73.9%
Address	Addis Ababa		158	66.4%
	Out of Addis Ababa		80	33.6%
	Primary		152	63.9%
	Secondary		4	1.7%
Duration of stay with chronic kidney disease	<1 year		63	26.5%
	1 – 3 years		77	32.4%
	3-5 years		50	21%
	5-10 years		48	20.2%
	Weight	Height	MAUC	BMI
Mean	24.25	1.24	16.43	15.23
Median	24.00	1.27	16.00	14.70
Std. Deviation	10.978	.257	4.187	2.940

### Hematologic profile of children with chronic kidney disease

The mean WBC count was 8.9 ± 3.32 (thousand/ L), with mean differential counts of monocyte 0.93 ± 3.02 (thousand/ L), eosinophil 0.3 ± 0.51 (thousand/ L), basophil 0.09 ± 0.24 (thousand/ L), neutrophil 4.59 ± 8.30 (thousand/ L), and lymphocyte 2.78 ± 1.62 (thousand/ L). About 164(68.9%) of the study participant have a normal range of white blood cell count, whereas 67 (28.2%) of them were with a high range of WBCs. Most prominently with a high percentage of differentials like lymphocytes 73(30.7%), monocyte 44 (18.5%), neutrophil 34(14.3%), basophil 23(9.7%) and eosinophil 22(9.2%) were found.

The mean RBC count was 4.73 ± 0.86 (million/L), of which 167(70.2%) of the children were in the normal range though 58(24.4%) had a low range of red blood cell count. RBC was found to be positively correlated with eGFR with a *P*-value of < 0.001. The average hemoglobin concentration was discovered to be 12.± 8 2.76(g/dL). The mean HCT was 38.2 ± 7.53%, which was significantly correlated with the eGFR with a *P*-value of 0.001. The mean red cell indices (MCV, MCH, and MCHC) were 80.3 ± 79.899 (fL), 27.6 ± 6.01 (Pg), and 32.9 ± 3.77 (g/dL), respectively.

 The mean PLT count of children with CKD was 349.3 ± 130.1(thousand/L). About 135(56.7%) of children with CKD have platelet count in the normal range whereas 41.2% have higher than normal range. Whereas a mean MPV of 9.03 ± 4.30 (fL) was determined which was significantly correlated with the eGFR with a *P* -value of < 0.001 and negative correlated with RBC, HGB and HCT with a *p*-value of 0.010, 0.006, and 0.004 (Tables 2, 3 and 4).

**Table 2:** Hematologic profile of children with chronic kidney disease on follow up in SPHMMC and TASH, Addis Ababa (*n*=238)

**Hematologic parameter**	**Mean **	**Std. deviation **
WBC(thousand/ µL)	8.9264	3.32235
RBC(million/ µL)	4.7305	.86673
HGB(g/dL)	12.8174	2.76429
HCT (%)	38.2839	7.53023
MCV(fL)	80.3181	7.89342
MCH(pg)	27.6937	6.01287
MCHC( g/dL)	32.9298	3.77578
PLT (thousand/ µL)	349.3445	130.17216
MPV(fL)	9.0301	4.30656
MONO (%)	8.3406	6.41159
EOS(%)	2.0072	2.41994
NEUT(%)	50.7218	18.85463
LYM(%)	37.1884	15.78053
BASO (%)	.5000	.87333
NEUT ( thousand/µL)	4.5964	8.30979
LYMPH( thousand/ µL)	2.7887	1.62244
EOS(thousand/µL)	.3057	.51134
MONO(thousand/µL)	.5000	3.02753
BASO(thousand/µL)	.0300	.24169
RDWSD	42.0000	10.03685

**Table 3:** Bivariate Correlational analysis of hematological parameters and eGFR of children with chronic kidney disease on follow up in SPHMMC and TASH, Addis Ababa (*n*=238)

	**eGFR**	
	**Pearson Correlation coefficient **	***P-value ***
RBC	.144	.000
HGB	.396	.004
HCT	.218	.001
PLT	-.082	.228
MPV	.153	.0001
	**MPV**	
	**Pearson Correlation coefficient **	***P-value ***
eGFR	.153	.0001
RBC	-.226	.010
HGB	-.181	.006
HCT	-.218	.004

**Table 4:** Hematologic profile of children with chronic kidney disease on follow up in St. Paul millennium medical college and Tikur Anbessa Hospital in Addis Ababa (*n*=238)

Hematologic profile	Normal range		Low range		High range	
	Freq.	Per.	Freq.	Per.	Freq.	Per.
WBC	164	68.9	7	2.9	67	28.2
MONO	166	69.7	28	11.8	44	18.5
EOSI	173	72.7	43	18.1	22	9.2
BASO	208	87.4	7	2.9	23	9.7
NEUT	156	65.5	48	20.2	34	14.3
LYMP	141	59.2	24	10.1	73	30.7
RBC	167	70.2	58	24.4	13	5.5
HGB	117	49.2	112	47.1	9	3.8
HCT	130	54.6	98	41.2	10	4.2
MCV	187	78.6	45	18.9	6	2.5
MCH	193	81.1	37	15.5	8	3.4
MCHC	184	77.3	44	18.5	10	4.2
RDW	177	74.4	1	.4	60	25.2
PLT	135	56.7	5	2.1	98	41.2

### Health conditions and co-morbidities of children with chronic kidney disease

The stages of CKD were determined and 81(34%) of children with CKD were in stage 1, 58(24.4%) were in stage 2, 63(26.5%) were in stage 3, 9(3.8%) were in stage 4, and 9(3.8%) were in stage 5. According to this study 128(53.8%), 30(12.6%), 14(5.9%), and 2(0.8%) respondents, the causes of their kidney disease were congenital abnormality, urinary tract infection, hypertension, and kidney stones, respectively. For 43(18.1%) of the study participants, co-morbidities such as congenital renal abnormalities with UTI, congenital renal abnormalities with hypertension, UTI with kidney stones, and hypertension with UTI contribute to the progression of their kidney problem into stages of CKD. In addition, 17(7.1%) of children have other health conditions such as congenital heart defect 9(3.8%), neurogenic bladder 3(1.3%), hydro ureter 2(0.8%), and hypertension 3(1.3%). Because of their kidney disease, 74(31.1%) of children have hypertension, for more than a year as 16(6.7%), 1–3 years 27(11.3%), 3–5 years 13(5.5%), and 5–10 years 18(7.6%). Despite this, only 16 (6.7%) of them monitor their hypertension at home.

 In this study, 39(16.4%) of the children were anemic and had lived with it for more than a year. And 24(10.1%) of them are currently being treated with anemia medications. Only 5 (2.1%) had been on dialysis before (Table 5). Regarding growth indicators, based on the WHO growth chart for BMI for age 5–19 years and MUAC for age less than 5 years, out of 176 children 5–17 years, 49(27.8%) have a BMI of < -1SD, 47(26.7%) have a normal BMI, and 32 have a low BMI (18.1% ) -2SD, and 19(10.7%) -3SD. For 176 children, 5–17 years, BMI was correlated with anemia with a *p*-value of 0.009 and medication with a *p*-value of 0.026. About 19 (36.5%) of children under the age of 5 have an arm circumference of − 2SD, 11 (21.1%) have a − 1SD, 7 (13.4%) have a normal MUAC, and 2 have a low MUAC (3.84%) − 3SD (Tables 6 and 7).

**Table 5:** Description of health condition and co-morbidities of children with chronic kidney disease on follow up in SPHMMC and TASH, Addis Ababa (*n*=238)

**Variable**		**Frequency **	**Percent **
Egfr	Stage 1(>90ml/min)	81	34%
	Stage 2(60-89ml/min)	58	24.4%
	Stage 3(30-59ml/min)	63	26.5%
	Stage 4(15-29ml/min)	9	3.8%
	Stage 5(15ml/min)	9	3.8%
Cause of Kidney disease	Congenital renal abnormalities	128	53.8%
	Hypertension	14	5.9%
	UTI	30	12.6%
	Kidney stones	2	0.8%
	UTI and kidney stones	8	3.4%
	Hypertension and UTI	6	2.5%
	Congenital abnormalities and UTI	24	10.1%
	Congenital abnormalities and HTN	5	2.1%
List of other co-morbidity	CHD(Congenital heart defect)	9	3.8%
	Neurogenic bladder	3	1.3%
	Hydro ureter	2	0.8%
	HTN	2	1.3%
Hypertension	Yes	74	31.1%
	No	164	68.9%
Anemia	Yes	39	16.4%
	NO	198	83.2%
Dialysis	Yes	5	2.1%
	No	233	97.9%

**Table 6:** Growth indicators based on WHO growth chart of children with chronic kidney disease on follow up in SPHMMC and TASH, Addis Ababa (*n*=238)

BMI For Age (5 – 19 years ) (*n* =176)			MUAC for age for less than 5 years of age (*n* = 52)	
	Freq.	Perc.	Freq.	Perc.
<- 3 SD	19	10.7%	2	3.84%
<- 2 SD	32	18.1%	19	36.5%
< - 1 SD	49	27.8%	11	21.1%
0 SD	47	26.7%	7	13.4%
>1 SD	17	9.65%	9	17.3%
>2 SD	11	6.25%	12	22.9%

**Table 7:** Bivariate Correlational analysis of Growth indicators and eGFR with other health conditions of children with chronic kidney disease on follow up in SPHMMC and TASH, Addis Ababa (*n*=238)

**Variable **	**Growth indicators **			
	**BMI**		**MUAC**	
	**P.C.C **	***P-value ***	**P.C.C **	***P-value ***
Longer stay With CKD			.251	.000
Hypertension			.327	.001
Anemia	.169	.009		
Using medication for Anemia	.145	.026		
Low platelet count			.161	.013
RDW			.149	.022
	**eGFR **			
	**P.C.C**		***P- Value***	
Hypertension	.197		.003	
Anemia	.211		.002	
Using medication for anemia	.208		.002	
Low range of RBC	.226		.001	
Low range of HGB	.181		.007	
Low range of HCT	.218		.001	

With *p*-values of 0.001, 0.007, and 0.001, a positive correlation were found between eGFR and a low range of RBC, HGB, and HCT. With a *p*-value of 0.002, this finding strengthens the relationship between anemia and the severity of the need for medication. With a *p*-value of 0.003, hypertension was also found to be significantly related to CKD stages based on eGFR (Table 7).

## Discussion

In this study, the hematological profile of children with chronic kidney disease was assessed on follow-up at SPHMMC and TASH, and it was discovered that 34% of stage1, 24.4% of stage2, 26.5% stage3, 3.8% stage4, and 3.8% stage5 of CKD based on their estimated glomerular filtration rate. With a *p*-value of 0.05, this finding was significantly correlated with having co-morbidities such as hypertension and anemia. Congenital abnormalities were the leading cause of kidney disease in children based on the study participant family and guardians’ respond. The study also found that having CKD for a longer period of time (> 5 years) and being hypertensive have correlation with growth indicator MUAC with *p*-values of < 0.001 and 0.001, respectively.

According to this study, 81 (34%) of the participants had stage 1 and 9 (3.8%) stage5 CKD; it’s consistent with a study conducted in Nigeria, which found that the majority of CKD patients (82.9%) were in stage1 [6]. In contrast, another North American study found that 68% of CKD patients were in stage 5 [7, 8]. Similarly, another study conducted in Cameroon found that the majority of CKD patients (81.3%) were in stage 5 and 18.3% were in stage 4 [9]. This contradicts our findings, which found that the majority of CKD patients 81 (34%) were in stage 1 and 9 (3.8%) were in stage 5. This disparity could be attributed to the late diagnosis of children with CKD, with the majority of patients having more than one indication, as well as the late presentation of sick children to the hospital.

The current study found that the total RBC count (4.73 ± 0.86 (million/L)), hemoglobin concentration (12.± 8 2.76(g/dL), and percentage hematocrit (38.2 ± 7.53%) had a positive correlation with CKD stages, indicating that the RBCs parameters were higher than the study conducted in Cameroon, which found that the hemoglobin level ranged from 5.26 to 10.1 gm/dl and the mean hemoglobin value was 7.68gm/dl [9]. Another study conducted in North America found that the mean hemoglobin level for the entire cohort was 11.81.8 g/dl, which is lower than the level found in this study. This difference could be attributed to the severity of chronic kidney disease and the large sample size/respondents involved in this study [10, 11]. In contrast, a similar study conducted in the United States and North America revealed that the mean ± SD of hemoglobin levels were 12.2 g/dl, ± 1.6 g/dl, and 12.5, ± 1.5 g/dl, which supports this study [12–14].

This study found that CKD stages were statistically significantly associated with low hemoglobin concentrations, which is similar to a North American study that found that CKD stages were statistically significant with anemia. In this study, the presence of anemia was 39(16.4%), which is lower than studies in North America, which revealed that 55% of study participants had anemia [8]. The presence of anemia increases as the stages of CKD rise, the risk of anemia in children with CKD increases as the GFR decreases, anti-hypertensive medications, and increased risk of hospitalization. Another multicenter study in the United States found that the prevalence of anemia was 47.7%, which is higher than this study. This disparity could be attributed to the large sample size/participation of respondents, the presence of comorbidity disease, and the severity of CKD [12].

The platelet count (349.3 ± 130.1(thousand/L)) in this study was significantly higher than in previous studies conducted in the United States, Cameroon, and Libya; these findings revealed that platelet counts were mostly normal [9, 15, 16].The reason for platelets count variation could be due to large sample size/respondents involvement, severity, and cause of CKD.

This study found that the leading and common cause of kidney disease in children is congenital abnormalities. On the contrary, the finding in Nigeria revealed primary kidney disease with acute glomerulonephritis, nephrotic syndrome and UTI was an important factor affecting the incidence of kidney dysfunction [6]. This difference could be due to the study being conducted in children hence that they are more susceptible to congenital problems. In addition, detection of AKI only when serum creatinine is elevated or has risen significantly low socioeconomic income, and lifestyle could contribute to this difference. On the contrary, another study conducted in North America revealed that elevated blood pressure was the most common cause of chronic kidney disease than a congenital abnormality. The reason for this variation is due to the absence of anti-hypertensive use, and the shorter duration of kidney.

This study found that 5(2.1%) of CKD patients began renal replacement therapy, which was lower than the study in Sub-Saharan Africa, Cameroon, and Nigeria. This contradicted a descriptive study in Sub-Saharan Africa, which discovered that 17% received renal replacement therapy, and a similar study in Nigeria, which discovered that 22 children required dialysis but only 15 (68.2%) received dialysis [6, 17]. Another similar study in Cameroon found that of 25 patients in renal recovery, 22 were complete, one was partial, and two were dialysis-dependent [9]. The most likely explanation for this finding is that TASH and SPHMMC have one of the few governmental dialysis centers in Ethiopia, which contributes to the higher prevalence of renal replacement therapy (RRT) initiation. Another study conducted in Cameroon and Nigeria found that 57.1% and 63.2% of CKD patients required dialysis [6, 9], respectively, which is higher than the current study. This disparity could be attributed to a lack of adapted dialysis material as well as financial constraints.

To the best of our knowledge we have tried to address the literature related to CKD, but there’s shortage of studies conducted on children CKD, as a result we could discuss all of our findings.

## Conclusion

The study found that the majority of study participants were in stages 1 to 3 based on their eGFR. Some hematological parameters of children with CDK showed variation from normal range with different level among parameters, thus these hematological parameters could contribute for CKD management besides other biochemical parameters. For instance, the following parameters could be indicators of the significance of hematological parameters for children with CKD; eGFR shows a positive correlation with a low range of RBC, HGB, and HCT with p-values of 0.001, 0.007, and 0.001, respectively. Besides, the mean MPV was positively correlated with eGFR with *p*-value 0.0001 whereas it was negatively correlated with RBC, HGB, and HCT with *p*-values of 0.010, 0.006, and 0.004, respectively. We recommend large scale study to further assess the hematological profiles among all age groups of CKD patients to identify the strength and values of these parameters to be used for CKD management.

## Data Availability

The datasets generated and/or analysed during the current study are not publicly available due [we do not have consent from all patients and ethical approval committees to publish this data] but are available from the corresponding author on reasonable request.

## Abbreviations

AKI: Acute kidney injury
BMI: Body mass index
CBC: Complete blood count
CKD: Chronic kidney disease
CRF: Chronic renal failure
EDTA: Ethylene diamine tetra acetic acid
EPO: Erythropoietin
ESR: Erythrocyte sedimentation rates
ESRD: End-stage renal disease
GFR: Glomerular filtration rare
eGFR: Estimated glomerular filtration rate
HCT: Hematocrit
HGB: Hemoglobin
LYM: Lymphocyte count
MCH: Mean corpuscular hemoglobin
MCHC: Mean corpuscular hemoglobin concentration
MCV: Mean corpuscular volume
MPV: Mean platelet volume
MUAC: Mid upper arm circumference
NEU: Neutrophil
PCC: Pearson correlation coefficient
PDW: Platelets distribution width
P-LCR: Platelet larger cell ratio
PLT: Platelets
RBC: Red blood cell
RDW: Red cell distribution width
RDW-SD: Red cell distribution width standard deviation
RRT: Renal replacement therapy
SD: Standard deviation
SPHMMC: St. Paul’s hospital millennium medical college
SPSS: Statistical package for the social science
TASH: Tikur Anbessa specialized hospital
UTI: Urinary tract infection
WBC: White blood cells
WHO: World health organization
